# Supplementary material for: Outer membrane lipoprotein NlpI scaffolds peptidoglycan hydrolases within multi‐enzyme complexes in Escherichia coli
Source: EMBO J. 2020 Feb 3;39(5):e102246. doi: 10.15252/embj.2019102246 (PMC7049810; doi:10.15252/embj.2019102246)
Supplement: Supplementary file 1 — Appendix [file EMBJ-39-e102246-s001.pdf]

APPENDIX

Outer membrane lipoprotein NlpI scaffolds peptidoglycan hydrolases within multi-enzyme complexes in *Escherichia coli*

Manuel Banzhaf[1,2*], Hamish C. L. Yau[3,9*], Jolanda Verheul[4*], Adam Lodge[3,7], George Kritikos[1], André Mateus[1], Baptiste Cordier[11], Ann Kristin Hov[1,8], Frank Stein[1], Morgane Wartel[1], Manuel Pazos[3], Alexandra S. Solovyova[5], Eefjan Breukink[10], Sven van Teeffelen[11], Mikhail M Savitski[1,6],Tanneke den Blaauwen[4#], Athanasios Typas[1,6#], Waldemar Vollmer[3#]

[1] European Molecular Biology Laboratory, Genome Biology Unit, Meyerhofstrasse 1, 69117 Heidelberg, Germany.

[2] Present address: Institute of Microbiology & Infection and School of Biosciences, University of Birmingham, Edgbaston, Birmingham, UK. B15 2TT.

[3] Centre for Bacterial Cell Biology, Biosciences Institute, Newcastle University, NE2 4AX, Newcastle upon Tyne, United Kingdom.

[4] Bacterial Cell Biology & Physiology, Swammerdam Institute for Life Sciences, Faculty of Science, University of Amsterdam; Science Park 904, 1098 XH Amsterdam, The Netherlands.

[5] Newcastle University Protein and Proteome Analysis, Devonshire Building, Devonshire Terrace, Newcastle upon Tyne, NE1 7RU, UK.

[6] European Molecular Biology Laboratory, Structural & Computational Unit, Meyerhofstrasse 1, 69117 Heidelberg, Germany.

[7] Present address: Iksuda therapeutics, The Biosphere, Newcastle upon Tyne, NE4 5BX.
[8] Present address: École polytechnique fédérale de Lausanne SV IBI-SV UPDALPE AAB 013, station 19, 1015 Lausanne, Switzerland.
[9] Present address: Newcastle University Faculty of Science, Agriculture and Engineering, NE1 7RX, Newcastle upon Tyne, United Kingdom.
[10] Membrane Biochemistry and Biophysics, Department of Chemistry, Faculty of Science, Utrecht University, Utrecht, Netherlands.
[11] Microbial Morphogenesis and Growth Lab, Institut Pasteur, Paris.

*These authors contributed equally to this work.

#Correspondence: T.denBlaauwen@uva.nl (T.dB.) typas@embl.de (A.T.), w.vollmer@ncl.ac.uk (W.V.)

Index

**Appendix Figure S1:**	*In vivo* and *in vitro* proteomic interaction assays link NlpI to several classes of PG hydrolases.
**Appendix Figure S2:**	NlpI dimerizes and interacts with several endopeptidases *in vitro*.
**Appendix Figure S3:**	Assaying for interactions between endopeptidases and with NlpI by Ni^{2+}-NTA pulldown assay.
**Appendix Figure S4:**	*In vitro* PG digestion assays.
**Appendix Figure S5:**	NlpI localizes along the cell envelope.
**Appendix Figure S6:**	Reconstitution of PG multi-enzyme complexes with PBP1A and LpoA.
**Appendix Figure S7:**	Initial fluorescence scans for MST experiments between PBP1A/LpoA with NlpI/EPases.
**Appendix Figure S8:**	PBP4 interacts simultaneously with NlpI and the PBP1A-LpoA synthase complex.
**Appendix Figure S9:**	PBP1A-LpoA activity is not significantly affected by the presence of MepS and/or NlpI.
**Appendix Figure S10:**	MepS*-NlpI does not affect the TPase activity of PBP1A-LpoA.
**Appendix Figure S11:**	BW25113 *vs.* BW25113Δ*nlpI* MP analysis.
**Appendix Figure S12:**	Expression of NlpI in a Δ*nlpI* strain partially restores wild-type morphology.
**Appendix Figure S13:**	MepS levels impact cell width.
**Appendix Table S1:**	Strain list
**Appendix Table S2:**	Plasmid list
**Appendix Table S3**:	Primer list

Fig. S1

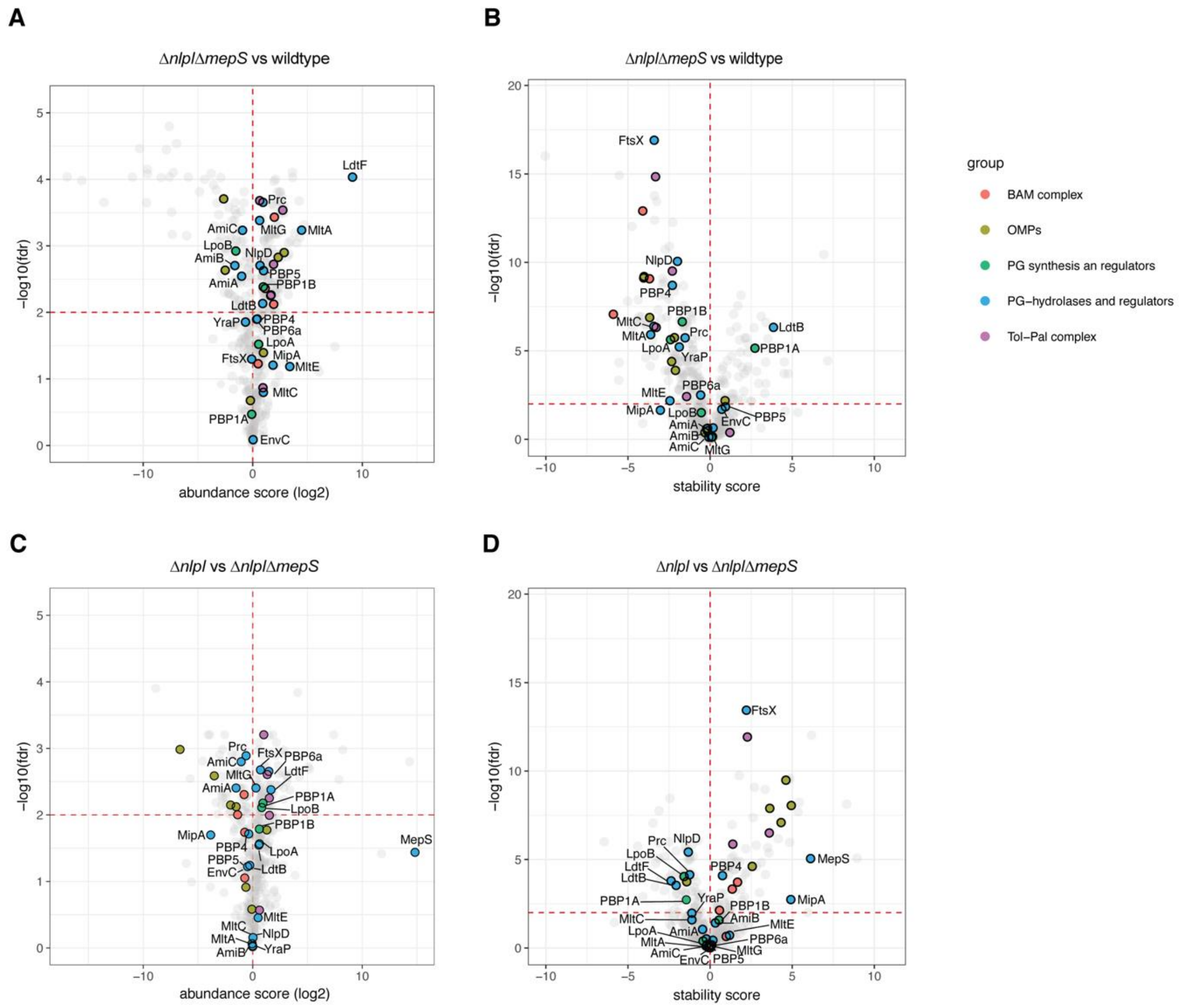

Fig. S1 – *In vivo* and *in vitro* proteomic interaction assays link NlpI to several classes of PG hydrolases.

A-B. Wild-type and Δ*nlpI*Δ*mepS* cells were heated at a range of temperatures and the soluble components were labelled, combined and quantified by LC-MS, using the published 2D-TPP protocol (Mateus *et al.*, 2018). The volcano plots of two replicates show protein abundance (a) and thermostability (b). A local FDR<0.01 was set as a threshold for significance. Abundance and stability scores of knocked out genes were discarded. Highlighted: outer membrane proteins (OMPs, light green), β-barrel assembly machinery (BAM proteins, red), PG synthases and regulators (green), PG hydrolases and regulators (blue) and the Tol-Pal complex (purple). Full results can be found in Tables EV 1 and 2.

C-D. 2D-TPP profiles of Δ*nlpI* compared to Δ*nlpI*Δ*mepS* cells. Shown in the volcano plots are changes in protein abundance (c) and thermostability (d). A local FDR<0.05 and a minimum absolute score of 10 were set as thresholds for significance. Highlighted: outer membrane proteins (OMPs, light green), β-barrel assembly machinery (BAM proteins, red), PG synthases and regulators (green), PG hydrolases and regulators (blue) and Tol-Pal complex proteins (purple). Full results can be found in Tables EV 1 and 2.

Fig. S2

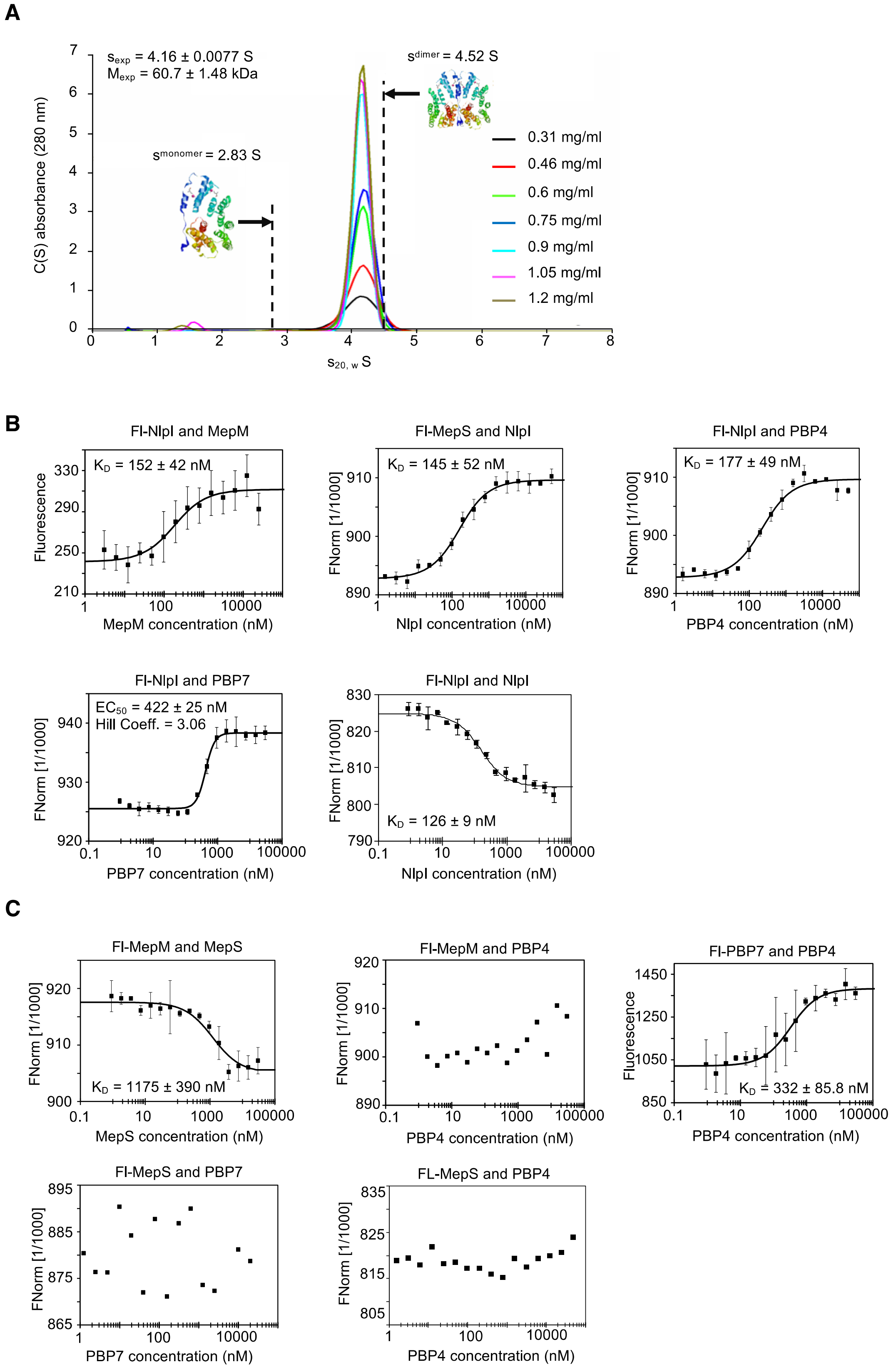

Fig. S2 – NlpI dimerizes and interacts with several EPases *in vitro*.
A. Dimer of NlpI detected by analytical ultracentrifugation (AUC). The soluble version of NlpI (residues 19-294) has a molecular weight of 30.35 kDa.

B. Microscale thermophoresis (MST) binding curves for interactions between fluorescently labelled MepS (fl-MepS) with unlabelled NlpI and fl-NlpI with unlabelled NlpI, MepM, PBP4 and PBP7. MST curves plotted are the mean data +/- SD of three independent experiments. Fl, fluorescently labelled; FNorm, normalized fluorescence. Ligand binding can affect thermophoresis differently; hence the binding of a ligand can enhance or reduce thermophoresis. This results in either a higher or lower FNorm in the bound state compared to the unbound state (Jerabek-Willemsen *et al.*, 2011).
C. Binding curves for MST assays between fl-MepM with MepS and PBP4, fl-PBP7 with PBP4 and fl-MepS with PBP4 and PBP7. MST curves plotted are the mean data +/- SD of three independent experiments. Fl, fluorescently labelled; FNorm, normalized fluorescence.

Fig. S3

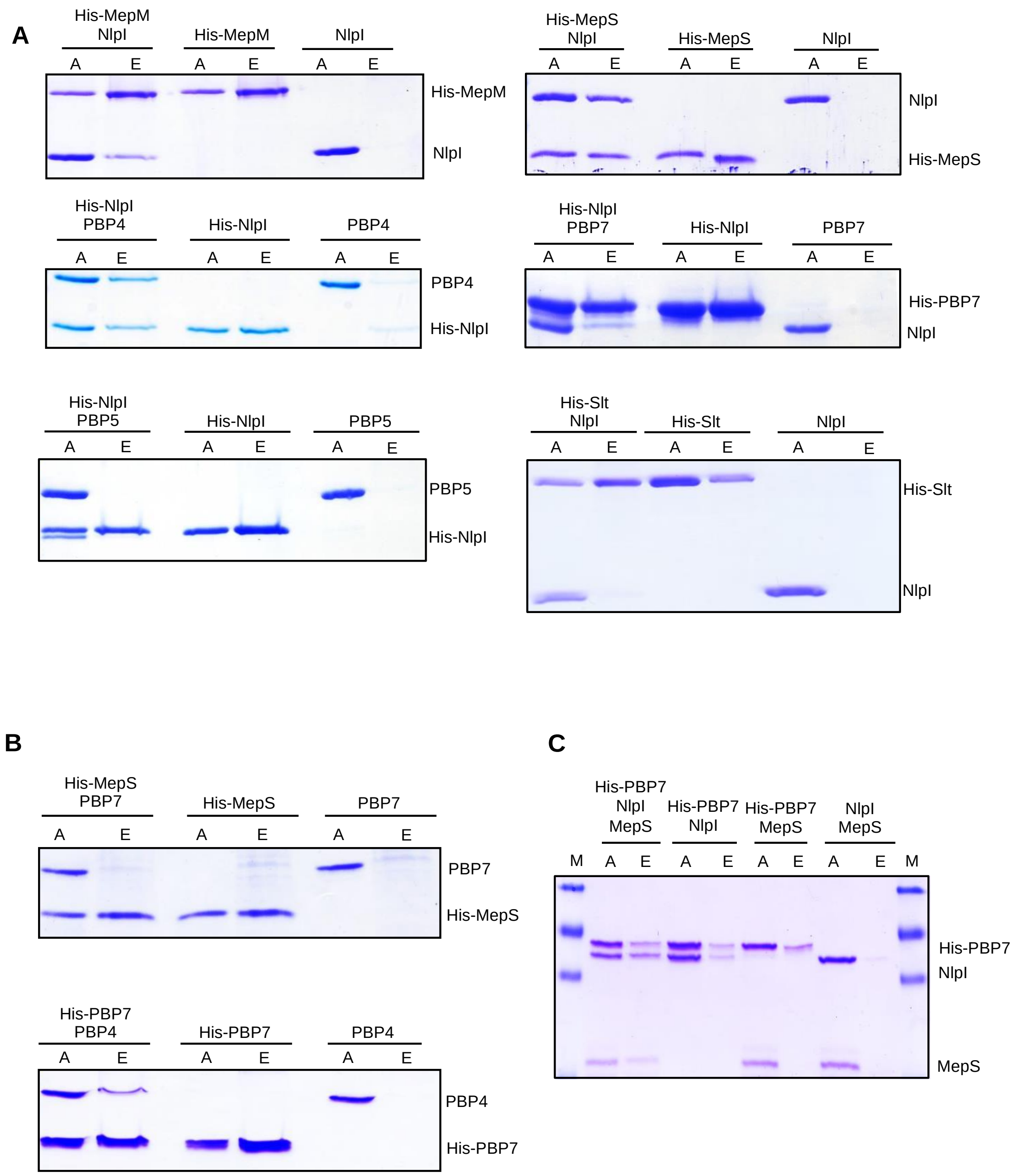

Fig S3. Assaying for interactions between EPases and with NlpI by Ni^{2+}-NTA pulldown assay.

A. SDS-PAGE (12-15%) analysis of applied (A) and eluted (E) samples from Ni^{2+}-NTA pull down assay of NlpI-EPase combinations. Equimolar concentrations of proteins for each respective assay (1 or 2 μM) were incubated with Ni^{2+}-NTA beads in combination or alone. Retention of tagless protein in the presence of His-tagged partner indicates an interaction. Tagless PBP5 is not retained in the presence of His-tagged NlpI nor is tagless NlpI retained by His-Slt.

B. SDS-PAGE (12-15%) analysis of applied (A) and eluted (E) samples from Ni^{2+}-NTA pull down assay of His-MepS with PBP7 and His-PBP7 with PBP4. Equimolar concentrations of proteins for each respective assay (1-2 μM) were incubated with Ni^{2+}-NTA beads in combination or alone.

C. SDS (15%) analysis of applied (A) and eluted (E) samples from Ni^{2+}-NTA pull down assay of His-PBP7 with NlpI (both 2 µM) and MepS (4 µM) in the presence of formaldehyde crosslinker. M, Marker.

Fig. S4

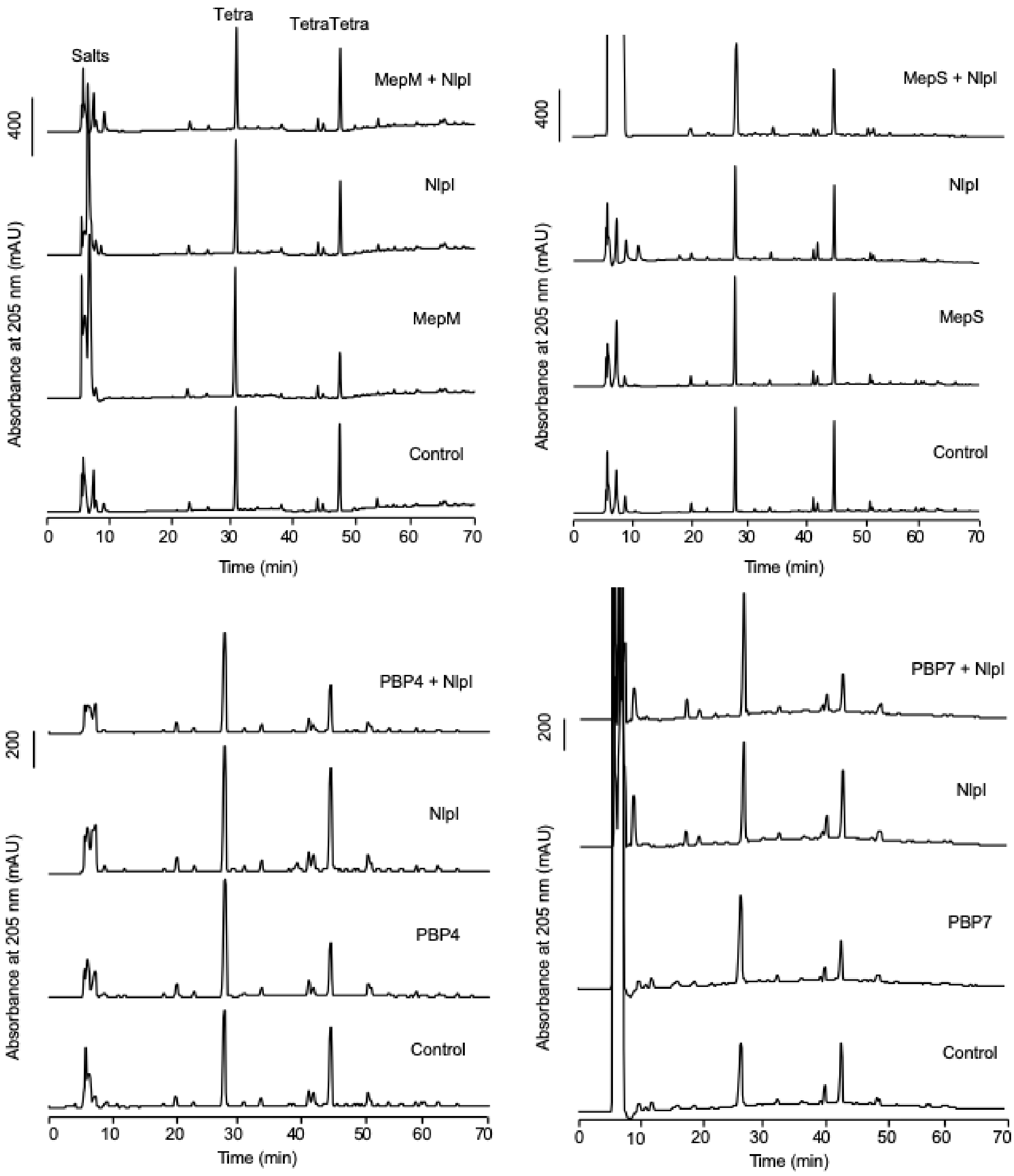

Fig. S4. *in vitro* PG digestion assays.
NlpI moderately affects the activity of MepM, but less so the activity of MepS, PBP4 and PBP7. Representative HPLC chromatograms of assays containing the respective EPase with or without NlpI. Control samples were incubated with no enzyme. Muropeptides were separated and the PG profiles determined as previously described (Glauner, 1988). Figure shows representative chromatograms of 3-6 independent experiments.

Fig. S5

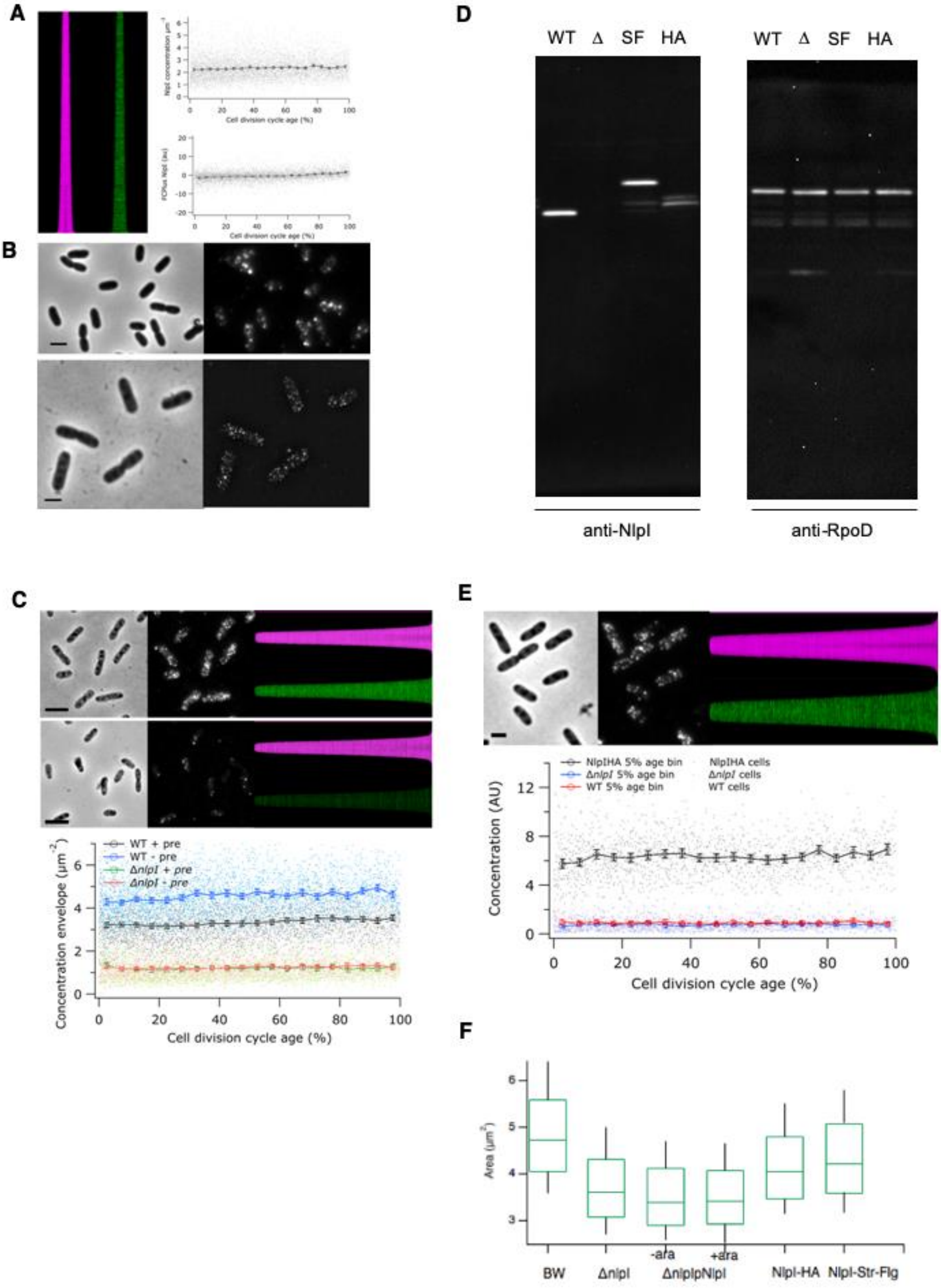
A
B
C
WT + pre
WT - pre
ΔnlpI + pre
ΔnlpI - pre
Concentration envelope (µm⁻²)
Cell division cycle age (%)
D
WT
Δ
SF
HA
anti-NlpI
anti-RpoD
E
NlpIHA 5% age bin
ΔnlpI 5% age bin
WT 5% age bin
NlpIHA cells
ΔnlpI cells
WT cells
Concentration (AU)
Cell division cycle age (%)
F
Area (µm²)
BW
ΔnlpI
-ara
+ara
ΔnlpIpNlpI
NlpI-HA
NlpI-Str-Flg

Fig. S5. NlpI localizes along the cell envelope.

A. MC4100 cells grown to steady state in minimal glucose medium at 28°C were immunolabeled with antibodies specific for NlpI. The map of diameters (magenta) and map of fluorescence (green) of NlpI localization where cells are sorted according to their cell length are shown. The concentration of NlpI in molecules per μm^{-2} wall area as function of the normalized cell division cycle age is constant. The grey dots are the values measured for the individual cells and the black markers are the 5% age bins with the 95 confidence error bars. The extra fluorescence at midcell compared to the rest of the cell (FCPlus) plotted as function of the normalized cell division cycle. NlpI is not specifically present at midcell (number of analyzed cells is 6087).

B. A high resolution fluorescence SIM image of BW23115 cells that were grown in LB at 37°C immunolabeled with anti-NlpI on the right and the corresponding Phase contrast image on the left is shown in the bottom panel. Scale bar equals 2 µm.

C. Wildtype BW25113 and the isogenic Δ*nlpI* strain were grown in TY at 37°C and fixed. The serum against NlpI was absorbed (pre) to the Δ*nlpI* cells and the remaining antibodies were used to label the wildtype strain (upper images) and another sample of the Δ*nlpI* cells (lower images). From left to right are shown: a phase contrast and the corresponding fluorescence image of the labelled cells and the map of diameters (magenta) and map of fluorescence (green) of the cells sorted according to ascending length. Scale bar equals 2 µm. Graph of the concentration NlpI (AU) in the envelope of the cells plotted against the cell division cycle time (%) are also shown. The dots are the data on the individual cells, whereas the markers are the 5% age bins with the 95 confidence error bars.

D. Western blot using anti-NlpI for constructs carrying different chromosomally tagged NlpI versions, and the wild-type and Δ*nlpI* controls. Cells were grown exponentially for 2 hours, harvested and adjusted to 2 µg total protein per lane. Samples were separated by SDS PAGE and visualised by Western Blot using primary antibodies against NlpI or RpoD, and anti-rabbit HRP. Samples are as followed: WT: BW25113; Δ: BW25113Δ*nlpI*::*kan* ; SF: BW25113*nlpI*::*strep*::*flag*::*kan*; HA: BW25113*nlpI*::*HA*::*frt*.

E. Wildtype BW25113 and the isogenic Δ*nlpI* strain and the same strain chromosomally expressing NlpI-HA were grown in TY at 37°C and fixed and labelled with anti HA. From left to right are shown: a phase contrast and the corresponding fluorescence image of the labelled cells and the map of diameters (magenta) and map of fluorescence (green) of the cells sorted with ascending length. Scale bar equals 1 µm. Also shown in panel D is a graph of the concentration HA-NlpI (AU) in the cells plotted against the cell division cycle time (%). The dots are the data on the individual cells, whereas the markers are the 5% age bins with the 95% confidence error bars.

F Cell morphology complementation assay of various NlpI versions. The area of the cells was determined from phase contrast images assuming the shape of the cells to consist of a cylinder with two half spheres as poles. BW is BW25113 the parental strain of the *nlpI* deletion strain Δ*nlpI*. Next sample is the Δ*nlpI* transformed with a plasmid that expresses NlpI under control of an arabinose inducible promotor (either without arabinose of after induction for 2 hours with arabinose (no difference between 01% or 0.2%). The last two samples are chromosomal fusions of NlpI with either a C-terminal HA-tag or a Strep-Flag tag.

Fig. S6

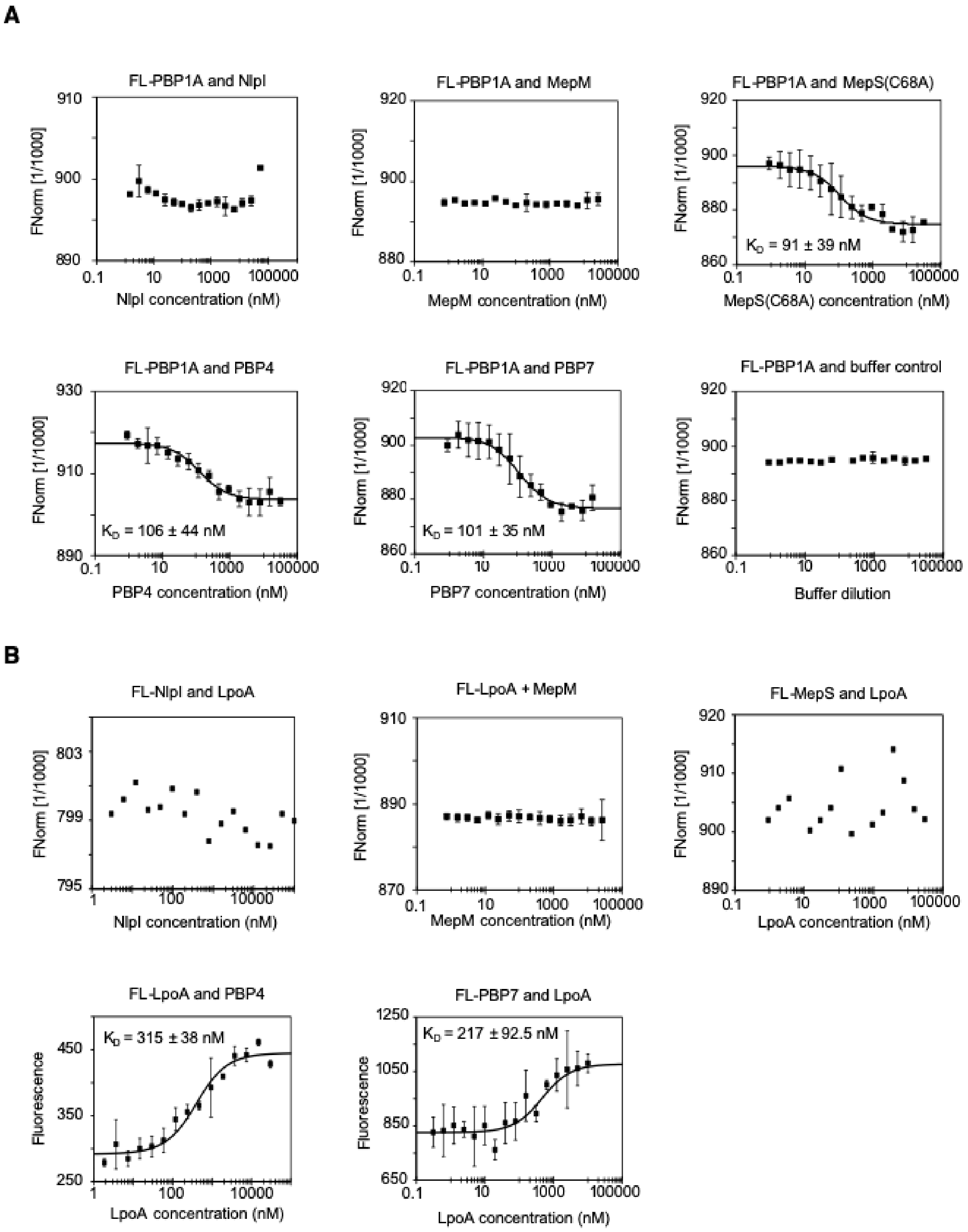

Fig. S6 – Reconstitution of PG multi-enzyme complexes with PBP1A and LpoA.
A. MST curves assaying for interaction between PBP1A with NlpI, MepM, MepS(C68A), PBP4, PBP7 and buffer control. A catalytically inactive version of MepS (MepS(C68A)) was used as unlabelled ligand in these experiments. MST curves plotted are the mean data +/- SD of three independent experiments. Fl, fluorescently labelled; FNorm, normalized fluorescence.
B. MST curves assaying for interaction between LpoA with NlpI, MepM, MepS, PBP4, PBP7. MST curves plotted are the mean data +/- SD of three independent experiments. Fl, fluorescently labelled; FNorm, normalized fluorescence.

Fig. S7

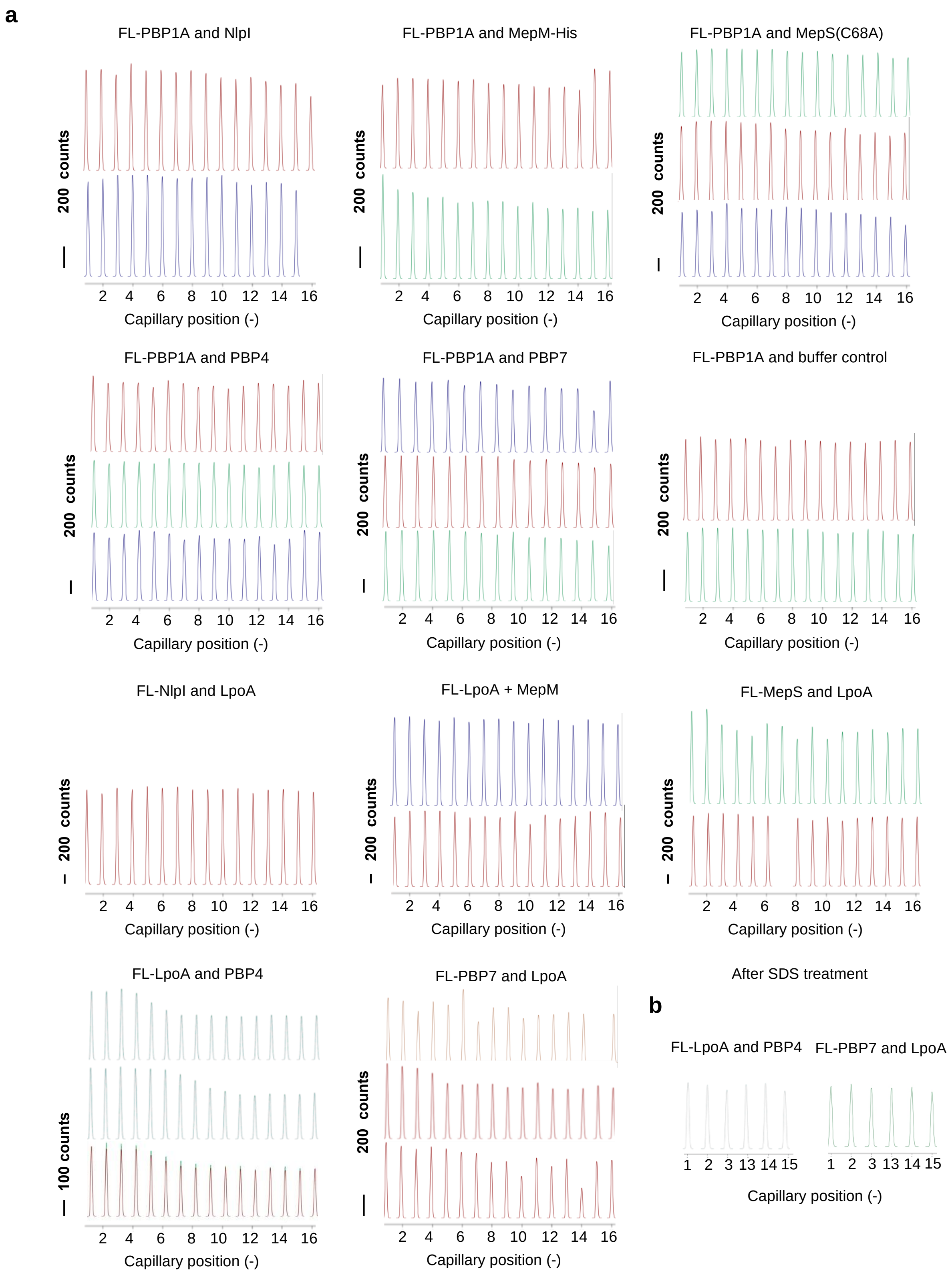

Fig. S7 – Initial fluorescence scans for MST experiments between PBP1A/LpoA with NlpI/EPases.

A. MST capillary scans showing initial fluorescence of respective reactions testing for interactions between PBP1A/LpoA against NlpI and EPases. Scale bar = 200 fluorescence counts.

B. Capillary scans for samples showing ligand concentration dependent changes in fluorescence were repeated after boiling samples in reducing agent and SDS to abolish ligand binding. The fluorescence reads for samples containing highest and lowest concentration of ligand were within the margin of error, suggesting that differences in initial fluorescence were due to ligand binding and not different Fl-protein concentration, validating the use of the raw fluorescence data to plot binding curves.

Fig. S8

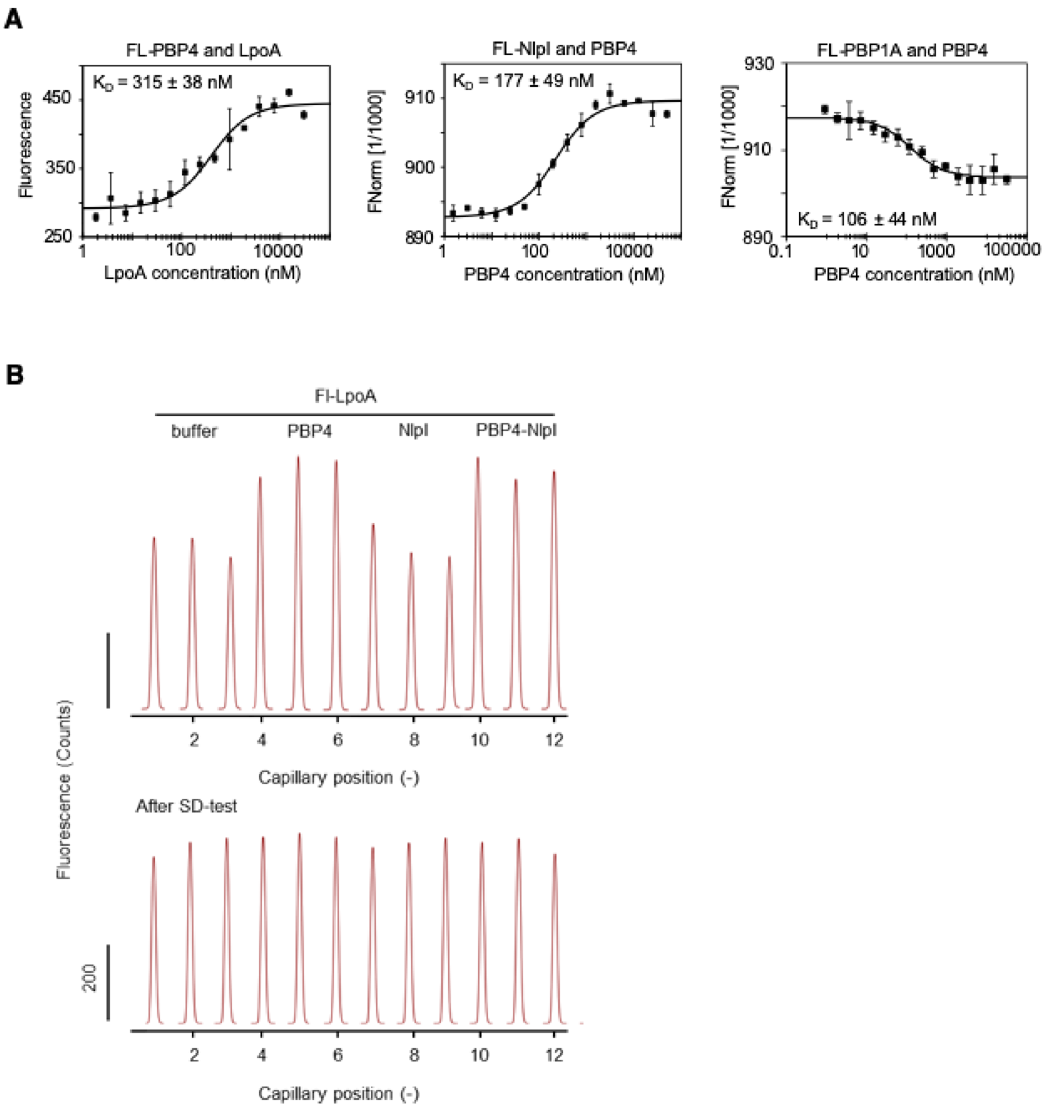

Fig. S8 – PBP4 interacts simultaneously with NlpI and the PBP1A-LpoA synthase complex.

A. MST interaction curves of PBP4 with PBP1A, LpoA and NlpI, respectively. The PBP4-LpoA interaction plot was already shown in Fig. S6B and is displayed here again for completeness. MST curves plotted are the mean data +/- SD of three independent experiments. Fl, fluorescently labelled; FNorm, normalized fluorescence.

B. Fixed concentration MST assays with LpoA-PBP4-NlpI showed differences in initial fluorescence. These differences were eliminated upon boiling of samples in SDS (which abolishes ligand binding), which suggests that the initial differences were due to ligand binding and not due to inaccurate pipetting, validating the use of the raw fluorescence data to plot binding curves.

Fig. S9

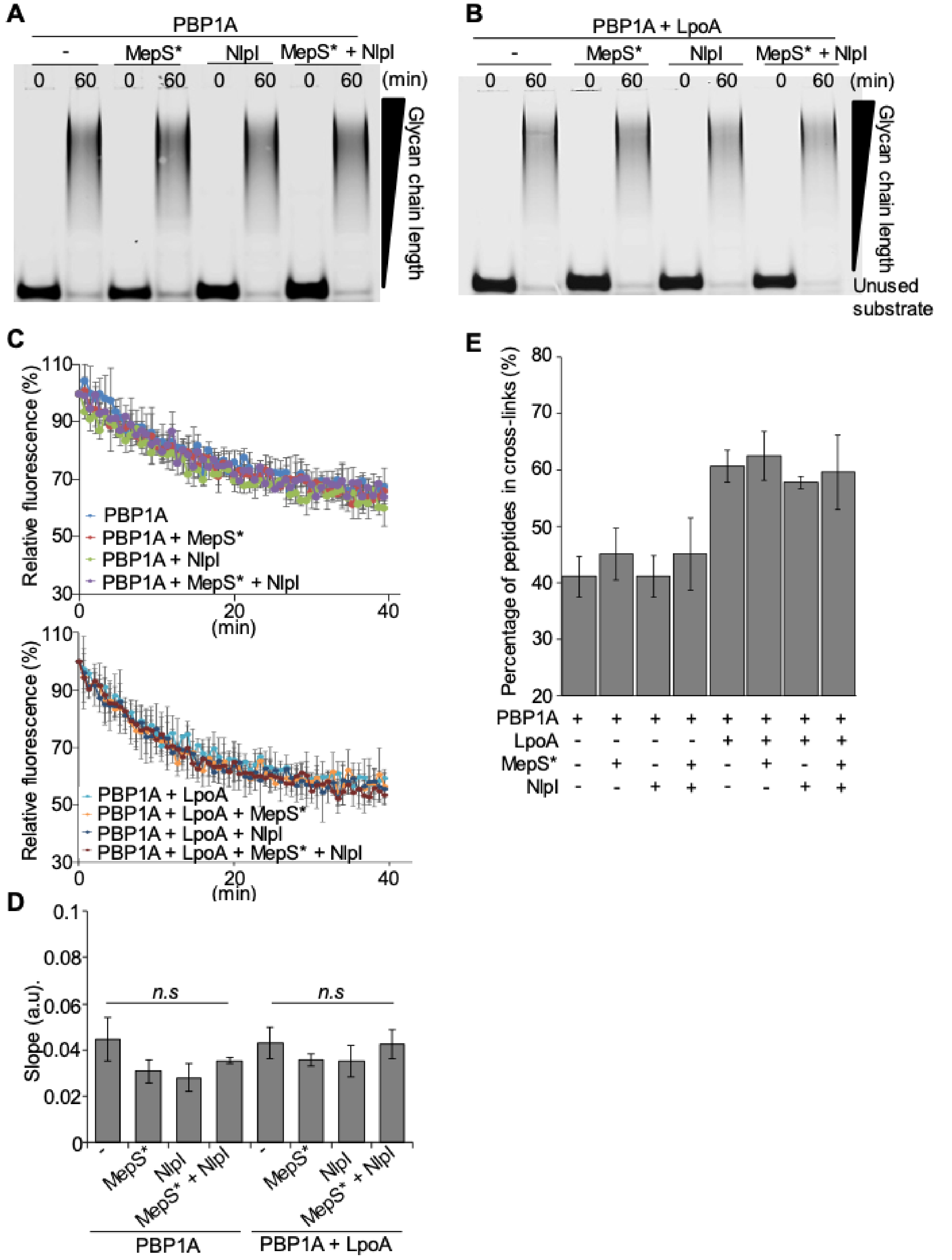

Fig S9 – PBP1A-LpoA activity is not significantly affected by the presence of MepS and/or NlpI.
ATTO[647]-Lipid II incorporation into glycan chains by **A** PBP1A or **B** PBP1A-LpoA, in the presence or absence of MepS^{C68A} (MepS*) and NlpI.
C. *in vitro* time course glycosyltransferase activity of PBP1A ± LpoA in the presence of MepS* and NlpI. Experiments were done in triplicate and error bars indicate standard deviation.

D. The initial gradient of slope (in panel C) against time is plotted as a graph to indicate lipid II incorporation. *n.s.,* not significant. (*p*-value < 0.05 was calculated using Students T-test)
E. Transpeptidase (TPase) activity of PBP1A ± LpoA in the presence or absence of MepS* and NlpI. Experiments were done in duplicate and error bars indicate range. Representative chromatograms shown in Fig. S10. and muropeptide quantifications are displayed in Table EV 6.

Fig. S10

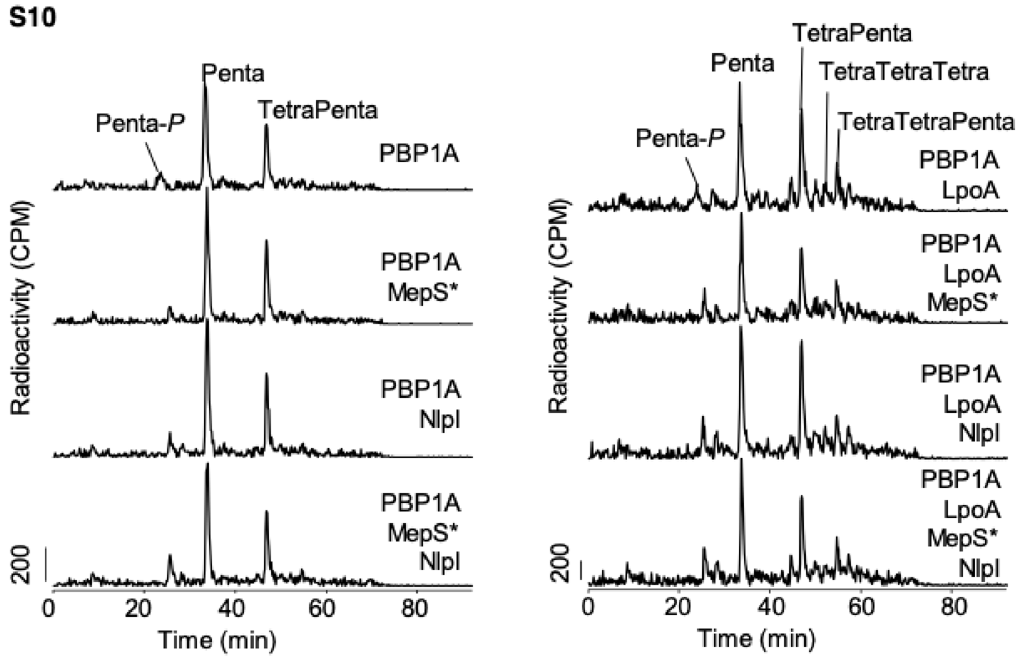

Fig S10 – MepS*-NlpI does not affect the TPase activity of PBP1A-LpoA. Representative chromatograms of an *in vitro* PG synthesis assays corresponding to Fig. S9e. CPM, counts per minute.

A

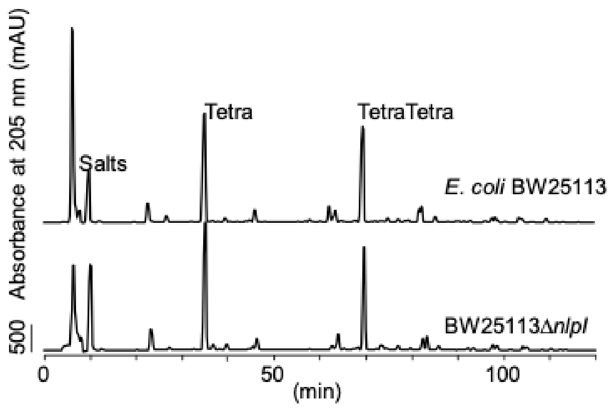

B

Muropeptide	Relative percentage (%) [1]	
	BW25113	BW25113Δ*nlpI*
Tri	5.3 ± 0.9	7.6 ± 0.6
TetraGly4	1.6 ± 0.2	0.8 ± 0.1
Tetra	42.5 ± 4.0	42.3 ± 1.5
Di	0.0 ± 0.0	1.6 ± 0.4
Penta	1.0 ± 0.0	2.8 ± 0.5
TetraTri(Dap)	2.3 ± 1.2	1.1 ± 0.4
TetraTetraGly	3.3 ± 0.4	3.4 ± 1.0
TetraTri	3.0 ± 0.8	0.0 ± 0.0
TetraTetra	32.0 ± 0.1	29.3 ± 0.7
TetraPenta	0.5 ± 0.7	1.2 ± 0.1
TetraTetraTriDap	0.0 ± 0.0	2.1 ± 1.3
TetraTetraTri	2.6 ± 0.3	3.0 ± 0.0
TetraTetraTetra	1.2 ± 0.4	1.0 ± 0.0
TetraTriAnh	0.5 ± 0.8	0.8 ± 0.3
TetraTetraAnh	0.9 ± 0.0	1.1 ± 0.0
TetraPentaAnh	2.3 ±0.2	1.1 ± 0.1
TetraTetraTetraAnh	1.0 ± 0.0	0.8 ± 1.1

[1] Values are mean ± variation of two independent experiments

C

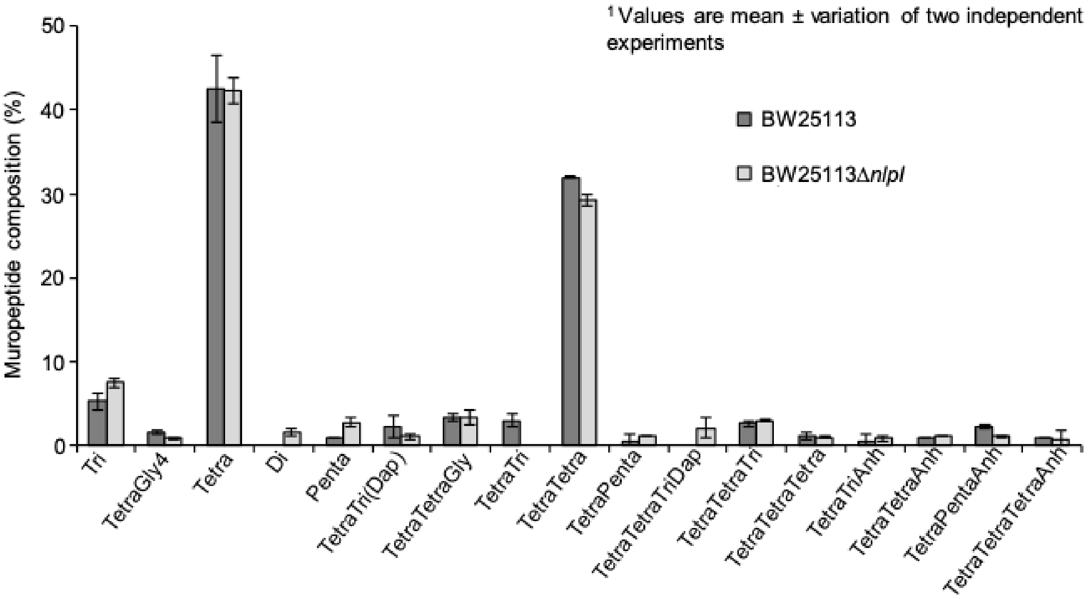

Fig. S11 – Loss of NlpI does not affect muropeptide composition of the cell.
A. HPLC chromatograms of muropeptides from *E. coli* strain BW25113 compared to BW25113Δ*nlpI*
B. Quantification of muropeptide composition in BW25113 and BW25113Δ*nlpI* (n=2)
C. graphical representation of muropeptide composition.

Fig. S12

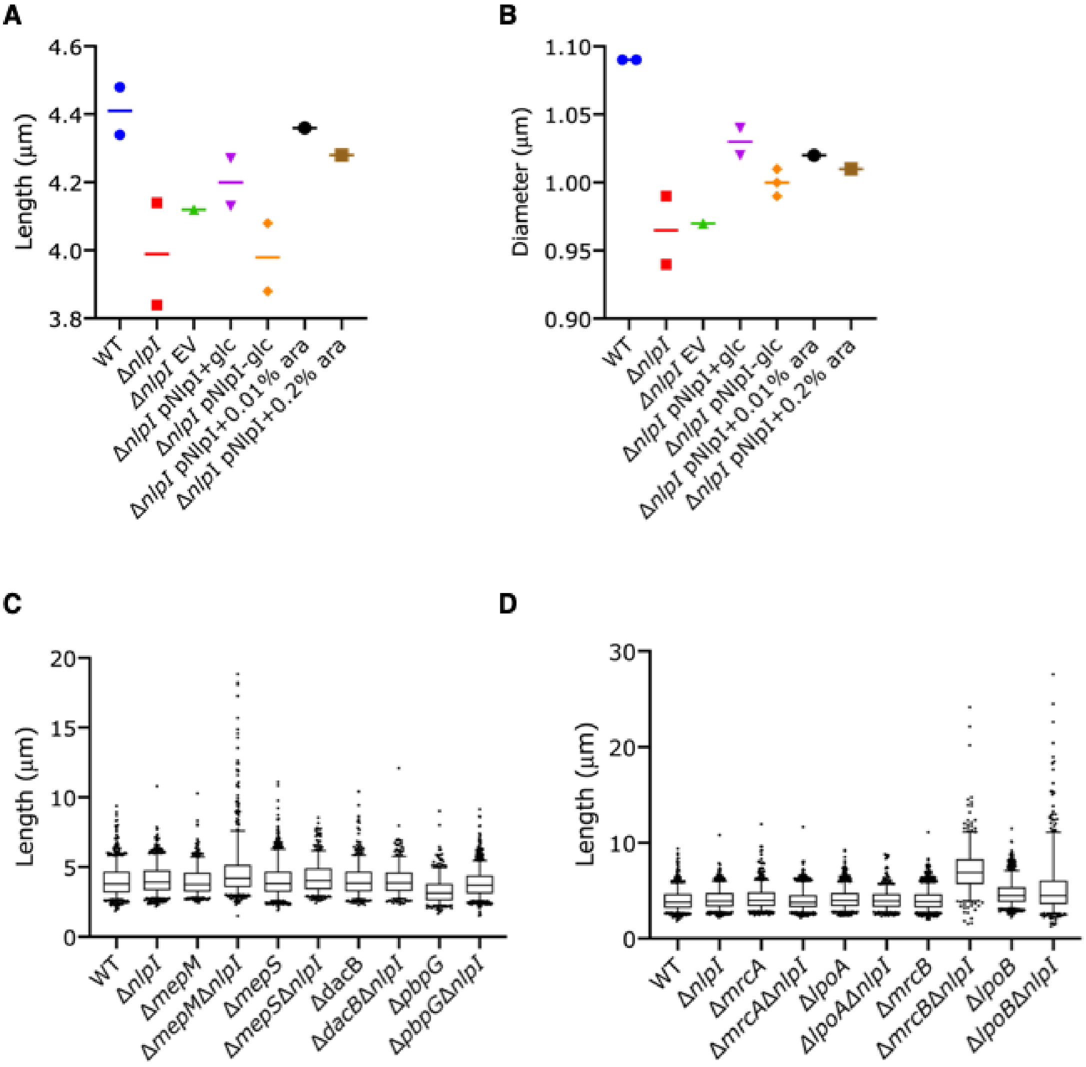

Fig. S12 – Expression of NlpI in a Δ*nlpI* strain partially restores wild-type morphology.

A and B. Cells were grown exponentially in TY at 37°C while the OD_{600} was kept below 0.3. Expression of the plasmid was suppressed by adding glucose, leakage allowed by growing without glucose or NlpI expression was induced by arabinose for 4 h. Each bar represents the average of one experiment with about 1000 cells (a) Distribution of cell length plotted against various growth conditions. (b) Distribution of cell diameter of cells grown under different conditions.

C. *nlpI* deletion changes the morphology of EPase-mutant strains. The graph shows the cell length of single and double deletions strains (800 < n < 2000 cells).

D. *nlpI* deletion exacerbates the morphological defects of the PBP1B/LpoB-mutant strains. The graph shows the cell length of single and double deletions strains (800 < n < 2000 cells).

Fig. 13

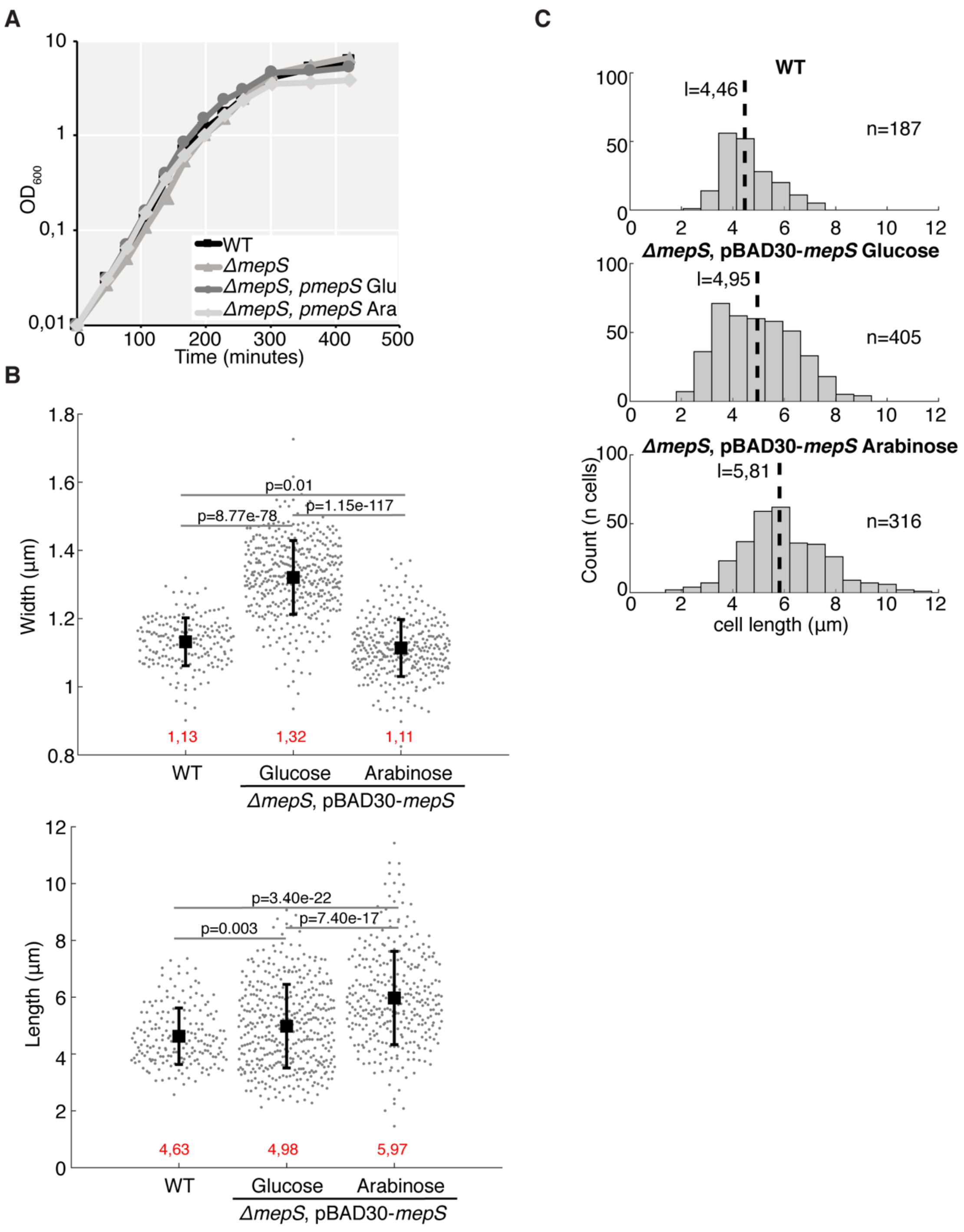

Fig. S13 – Effect of MepS expression on cell shape.
A. Variations of MepS levels have no impact on growth rate. Strains were grown in LB at 30°C. For the strain Δ*mepS*, pBAD30-*mepS*, the medium was supplemented with carbenicillin (100 µg/mL) and 0,2% glucose to repress *mepS* or 0,2% L-arabinose for induction. OD_{600} was measured over time.
B. Alternative representation of Fig. 3f and S13c. Points indicate single-cell width (left plot) and length (right plot). Squares indicate the population average, and error bars indicate the standard deviation between single cells. The calculated p-value (p) between two populations is indicated and the value of the mean width and length is indicated in red (□m). The number of cells (n) measured for the WT strain is 187,

n=405 for the Δ*mepS*, pBAD30-*mepS* strain grown with glucose and n=316 for the Δ*mepS*, pBAD30-*mepS* strain grown with L-arabinose.

C. Distribution of mean cell length. For each strain and condition, the value of the median length (l) and the number of cells (n) is indicated.

Appendix Tables:

Appendix table S1 – Strain list

***E. coli* strain**	**Properties / Relevant features**	**Source**
BL21(DE3)	Overexpression strain F- *ompT, dcm lon hsdS hsdSB*(rB-mB-) λ(DE3)	Novagen
MC1061	Laboratory strain araD139 Del(*araA-leu*)7697 Del(lac)X74 *galK16 galE15*(GalS) *lambda- e14- mcrA0 relA1 rpsL150(strR) spoT1 mcrB1 hsdR2*	(Datsenko & Wanner, 2000)
LMC500 (MC4100)	F-, araD139, Δ(argF-lac)U169, deoC1, flbB5301, lysA1, ptsF25, rbsR, relA1, rpsL150	(Taschner et al.,1988)
NT10001	BW25113 Keio parental strain F-, DE(araD-araB)567, lacZ4787(del)::rrnB-3, LAM-, rph-1, DE(rhaD-rhaB)568, hsdR514	(Datsenko & Wanner, 2000)
NT10020	BW25113 Δ*ydho*::kan	This study
NT10023	BW25113 Δ*dacB*::kan	This study
NT10067	BW25113 Δ*mepA*::kan	This study
NT10068	BW25113Δ*pbpG*::kan	This study
NT10075	BW25113 Δ*yebA*::kan	This study
NT10210	BW25113 Δ*nlpI*::tet	This study
NT10258	BW25113 Δ*spr*Δ*nlpI*::tet	This study
NT10259	BW25113 Δ*yebA*Δ*nlpI*::tet	This study
NT10260	BW25113 Δ*ydhO*Δ*nlpI*::tet	This study
NT10261	BW25113 Δ*mepA*Δ*nlpI*::tet	This study
NT10262	BW25113 Δ*pbpG*Δ*nlpI*::tet	This study
NT10263	BW25113 Δ*dacB*::kanΔ*nlpI*::tet	This study
NT1051	BW25113 Δ*nlpI::tet*Δ*spr::kan*	This study
NT10502	BW25113 Δ*spr*::kan	This study
NT10507	BW25113 Δ*mrcA*::kan	This study
NT10524	BW25113 Δ*mrcB*::cat	This study
NT10548	BW25113 Δ*mrcA*::kanΔ*nlpI*::tet	This study
NT10620	BW25113 Δ*mrcB*::kan	This study
NT10621	BW25113 Δ*lpoA*::kan	This study
NT10622	BW25113 Δ*lpoB*::kan	This study
NT10628	BW25113 Δ*mrcB*::kanΔ*nlpI*::tet	This study
NT10629	BW25113 Δ*lpoA*::kanΔ*nlpI*::tet	This study
NT10630	BW25113 Δ*lpoB*::kanΔ*nlpI*::tet	This study
NT10678	BW25113 Δ*nlpI*::tetΔ*lpoB*::kan	This study
NT10679	BW25113 Δ*nlpI*::tetΔ*mrcA*::cat	This study
NT10680	BW25113 Δ*nlpI*::tetΔ*mrcB*::cat	This study
NT10681	BW25113 Δ*nlpI*::tetΔ*lpoA*::cat	This study
NT10698	BW25113 Δ*nlpI*::tetΔ*dacB*::kan	This study

NT10699	BW25113 Δ*nlpI*::tetΔ*pbpG*::kan	This study
NT10700	BW25113 Δ*nlpI*::tetΔ*mepA*::kan	This study
NT10701	BW25113 Δ*nlpI*::tetΔ*ydhO*::kan	This study
NT10702	BW25113 Δ*nlpI*::tetΔ*yebA*::kan	This study
NT10703	BW25113 Δ*nlpI*::tetΔ*spr*::kan	This study
NT11148	BW25113 *nlpI*-HA	This study
NT11307	BW25113 *nlpI*-SF	This study
MG1655	F- lambda- *ilvG*- *rfb*-50 *rph*-1	bioRxiv 769786; doi: https://doi.org/10.1101/769786
E54	MG1655 Δ*mepS*	bioRxiv 769786; doi: https://doi.org/10.1101/769786
B195	Δ*mepS*, pBAD30-*mepS*	bioRxiv 769786; doi: https://doi.org/10.1101/769786

Appendix Table S2 – Plasmid list

Plasmid	Properties / Relevant features	Source
pET21b-MepS-His	MepS residue 28-188, C-terminal His-tag, Amp^R	(Singh *et al.*, 2012)
pET21b-PBP4Δ1-60	Native PBP4 lacking residues 1-60, Amp^R	Manuel Pazos (Vollmer group)
pET28a-His-MepM	MepM residues 40-440, N-terminal His-tag, Kan^R	(Singh *et al.*, 2012)
pET28a-His-NlpI	Soluble NlpI construct, N-terminal His-tag, Kan^R	Manuel Banzhaf (EMBL, Heidelberg)
pET28a-His-LpoA	Soluble LpoA construct, N-terminal His-tag, Kan^R	(Jean *et al.*, 2014)
pET28a-His-PBP7	PBP7, N-terminal His-tag, Kan^R	Ann-Kristin Hov (Vollmer group)
pTK1A-His	Full length His-PBP1A, Kan^R	(Born *et al.*, 2006)
pKD13	KmR used to amplify the kanamycin cassette	Laboratory collection
pCB112	cat lacIq P_{lac} ::lacZ	(Paradis-Bleau et al, 2014)
pJSP1	KmR used to amplify the Strep-Flag tag and the kanamycin cassette	Laboratory collection

Appendix Table S3 – Primer list

Name	Sequences of primers (5′ – 3′)
74-NlpI-HA-O1	GCCAGGACCAAGATGACCTGGCAGAATCGGACCAGCAATACCCATACGACGTCCCAGACTACGCTTACCCATACGACGTCCCAGACTACGCTTAAgtgtaggctggagctgcttc
87-NlpI-HA-O2	CGGGCTGATGTGTACGTCAGattccggggatccgtcgacc
175-NlpI-SF-O1	TATCGCTCCTGGGCCAGGACCAAGATGACCTGGCAGAATCGGACCAGCAAGGCGGCGGCTGGTCACACCCGCAGTTTG
176-NlpI-SF-O2	TAAGGTGATGGCAATCAAAAAAGATTACGGGCTGATGTGTACGTCAGcatatgaatatcctccttag

References

Born, P., Breukink, E., and Vollmer, W. (2006). In vitro synthesis of cross-linked murein and its attachment to sacculi by PBP1A from *Escherichia coli. J Biol Chem* **281**, 26985-26993.

Datsenko, K.A., and Wanner, B.L. (2000). One-step inactivation of chromosomal genes in *Escherichia coli* K-12 using PCR products*. Proc Natl Acad Sci U S A* **97**, 6640-6645.

Glauner, B. (1988). Separation and quantification of muropeptides with high-performance liquid chromatography*. Anal Biochem* **172**, 451-464.

Jean, N.L., Bougault, C.M., Lodge, A., Derouaux, A., Callens, G., Egan, A.J., Ayala, I., Lewis, R.J., Vollmer, W., and Simorre, J.P. (2014). Elongated structure of the outer-membrane activator of peptidoglycan synthesis LpoA: implications for PBP1A stimulation*. Structure* **22**, 1047-1054.

Jerabek-Willemsen, M., Wienken, C.J., Braun, D., Baaske, P., and Duhr, S. (2011). Molecular interaction studies using microscale thermophoresis*. Assay and Drug Development Technologies* **9**, 342-353.

Mateus, A., Bobonis, J., Kurzawa, N., Stein, F., Helm, D., Hevler, J., Typas, A., and Savitski, M.M. (2018). Thermal proteome profiling in bacteria: probing protein state *in vivo. Molecular Systems Biology* **14**.

Singh, S.K., SaiSree, L., Amrutha, R.N., and Reddy, M. (2012). Three redundant murein endopeptidases catalyse an essential cleavage step in peptidoglycan synthesis of *Escherichia coli* K12*. Mol Microbiol* **86**, 1036-1051.

Taschner, P.E., Huls, P.G., Pas, E., and Woldringh, C.L. (1988). Division behavior and shape changes in isogenic *ftsZ, ftsQ, ftsA, pbpB,* and *ftsE* cell division mutants of *Escherichia coli* during temperature shift experiments*. J Bacteriol* **170**, 1533-1540.
